# Investigating the relationship between prenatal alcohol exposure and children’s behavioural and emotional development: analysis of the Growing Up in New Zealand study

**DOI:** 10.1093/alcalc/agae029

**Published:** 2024-04-27

**Authors:** Joanna Ting Wai Chu, Jessica McCormack, Yannan Jiang, Daniel Walsh, Holly Wilson, Samantha Marsh, Fiona Langridge, Chris Bullen

**Affiliations:** Social and Community Health, School of Population Health, The University of Auckland, Private Bag 92019, Auckland Mail Centre, Auckland 1142, New Zealand; Centre for Arts and Social Transformation, Faculty of Arts and Social Work, The University of Auckland, Private Bag 92019, Auckland Mail Centre, Auckland 1142, New ZealandNew Zealand; Food Science, University of Otago, PO Box 56, Dunedin 9054, New Zealand; Social and Community Health, School of Population Health, The University of Auckland, Private Bag 92019, Auckland Mail Centre, Auckland 1142, New Zealand; Statistics, Faculty of Science, The University of Auckland, Private Bag 92019, Auckland Mail Centre, Auckland 1142, New Zealand; Social and Community Health, School of Population Health, The University of Auckland, Private Bag 92019, Auckland Mail Centre, Auckland 1142, New Zealand; Social and Community Health, School of Population Health, The University of Auckland, Private Bag 92019, Auckland Mail Centre, Auckland 1142, New Zealand; Department of Paediatrics, Child and Youth Health, The University of Auckland, Private Bag 92019, Auckland Mail Centre, Auckland 1142, New Zealand; National Institute for Health Innovation, School of Population Health, The University of Auckland, Private Bag 92019, Auckland Mail Centre, Auckland 1142, New Zealand

**Keywords:** prenatal alcohol exposure (PAE), neurocognitive outcomes, behavioural, emotional development

## Abstract

**Aims:**

To examine the relationship between prenatal alcohol exposure (PAE) and children’s behavioural and emotional development in a large generalizable sample of women and their children in Aotearoa New Zealand.

**Methods:**

Using data from the Growing Up in New Zealand longitudinal cohort, we investigated the relationship between maternal PAE and behavioural and emotional development in 8-year-old children. We explored secondary outcomes including measures of language, executive function, academic achievement, and adaptive behaviour.

**Results:**

We found no significant differences in the measures of behavioural and emotional development in children 8 years old based on alcohol consumption. No significant differences in behavioural and emotional development were found based on amount of PAE and when PAE occurred, despite controlling for a range of potential confounding factors, such as neighbourhood deprivation and maternal health measures. PAE was associated with significantly higher scores for parent-rated oral language indicating better oral language. In Māori mothers, PAE was significantly associated with an increased risk of higher scores on two of the Strengths and Difficulties Questionnaire subscales.

**Conclusions:**

We did not find an association between PAE and behavioural and emotional development in children aged 8 years. PAE and behavioural and emotional development are difficult to measure accurately, and the moderating variables between them are complex. Future analyses will require larger cohorts of mothers and their children using precise measures of PAE and outcomes to enable more precise estimates of association.

## Teaser

**Short Summary:** This study explores the relationship between prenatal alcohol exposure and behavioural and emotional development in a large representative cohort. Although we did not find an association between prenatal alcohol exposure and behavioural and emotional development, this could be due to moderating variables or difficulty measuring prenatal alcohol exposure.

## Introduction

In Aotearoa New Zealand (NZ), guidelines for alcohol use in pregnancy advise that there is no known safe level during pregnancy (Ministry of Health 2018). Prenatal alcohol exposure (PAE) may result in neurodevelopmental impairments, ranging from domain-specific deficits to global impairments with lifelong effects (Mattson et al. 2019). Globally, approximately 9.8% of pregnancies are exposed to alcohol (Popova et al. 2017). In NZ, 71% of women consumed alcohol before becoming pregnant, while 23% reported drinking alcohol during pregnancy and 13% after the first trimester (Rossen et al. 2018). The odds of consuming any alcohol during pregnancy were significantly higher for European or Māori (Indigenous people) women (Rossen et al. 2018). Māori and Pacific families are disproportionally affected due to the historical trauma and political, socioeconomic, and health conditions that contribute to inequitable health outcomes (Walker 2019; Espiner et al. 2022).

PAE may cause a wide range of adverse neurological and physical effects, collectively known as fetal alcohol spectrum disorder (FASD) (Harding et al. 2019). The relationship between PAE and neurological outcomes is complex as some studies show an adverse effect and others do not (Chu et al. 2022b). One of these domains that children with PAE or FASD can experience difficulties with is behavioural and emotional development (Cook et al. 2016). Typically, PAE has adverse consequences on behavioural and emotional development, but the relationship varies with the timing and amount of alcohol exposure, and the sex of the child (Chu et al. 2022). Exposure to large amounts of alcohol and binge drinking were the most consistently reported exposures with consequences on externalizing, internalizing behaviour and conduct problems (Skogerbo et al. 2013; Sayal et al. 2014; Schoeps et al. 2018; Chu et al. 2022). However, other studies found no effect of PAE on behavioural and emotional development (D'souza et al. 2019). Some of this variation could be due to the child’s sex. In one study, PAE had adverse outcomes on conduct problems for girls only (Sayal et al. 2014), whereas another found no adverse outcomes for girls (Niclasen et al. 2014). Several methodological factors may contribute to this discrepancy. One of these is the failure to control for unmeasured confounding variables in the prenatal environment (Chu et al. 2022) that could impact the relationship between PAE and behavioural and emotional development, such as traumatic events (Price et al. 2017). In this study, we explored the relationship of PAE and behavioural and emotional development in NZ children using data from the *Growing Up in New Zealand* (GUiNZ) cohort study’s 8-year dataset. The GUiNZ is a longitudinal study of children born in the Auckland region between 2009 and 2010. The 8-year dataset was chosen because the neurodevelopmental outcomes PAE may affect may be evident in children at 8 years old.

## Methods

We analysed data from the GUiNZ study to examine the relationship between PAE and children’s neurocognitive development outcomes at 8 years.

### Study population

The GUiNZ cohort is a longitudinal study of children whose mothers were recruited from NZ prospectively between 2008 and 2010. The methods of the GUiNZ study are described on their website (http://www.growingup.co.nz/en/about-the-study.html). Information was collected from pregnant mothers and their partners from pre-birth and continuing up to when the children were 8 years of age. Pregnant women were eligible to participate in the study if they had an expected delivery date between 25 April 2009 and 25 March 2010. The sample is generalizable of all NZ births in 2007–2010 (Morton et al. 2012). In total, 6822 pregnant women enrolled in the GUiNZ study (Morton et al. 2012); the cohort consists of 6853 children, of which 6643 were singleton births. All multiple birth babies were excluded from analysis due to the small number from the cohort.

### Measurement of prenatal alcohol intake

Antenatal interviews took place during the last trimester of pregnancy (*n* = 5668) or postnatally (*n* = 1154). Women were asked to estimate the average number of NZ standard drinks (i.e. 10 g of pure alcohol) consumed per week for the 3 months before pregnancy (T1), for the first 3 months of pregnancy (T2), and after the first 3 months of pregnancy (T3). Responses were given in the number of drinks per week and categorized in the dataset as follows: did not drink, less than 1 drink per week, 1–3 drinks, 4–19 drinks, and ≥20 drinks per week.

We classified women into three exposure categories: (a) Non-drinker, if they did not consume alcohol before or during pregnancy; (b) Abstainer, if they consumed alcohol before but not during pregnancy; and (c) Alcohol Exposed, if they reported consuming alcohol at any time during the pregnancy (i.e. T2 or T3). We distinguished Abstainer and Non-drinker due to differences in socioeconomic determinants in health between these groups that may impact upon neurodevelopmental outcomes (Alvik et al. 2013).

### Primary outcome

The primary outcome was emotional and behavioural development measured on the Strengths and Difficulties Questionnaire (SDQ) in children aged 8 years. The SDQ (Goodman 1997) is a self-report questionnaire completed by a parent or teacher that measures emotional and behavioural problems. The SDQ contains 25 items, with 5 subscales, hyperactivity, conduct problems, emotional problems, peer problems, and prosocial behaviour (see Appendix Table S1). The SDQ Total Differences Score has been validated across a variety of age groups and populations (up to 17 years), including New Zealand children <5 years (Kersten et al. 2018). Several longitudinal cohort studies have used the SDQ as a measure of emotional and behavioural difficulties (Skogerbo et al. 2013; Niclasen et al. 2014; Schoeps et al. 2018; D'souza et al. 2019). The SDQ was completed by mothers when their children were aged 2 (DCW2), 5 (DCW 5), and 8 years (DCW 8), with parents completing the SDQ for ages 2–4 at age 2 and 4–17 at ages 5 and 8. Analyses were conducted on the Total Difficulties Score and each of the five subscales (Goodman 2001).

### Secondary outcomes

Secondary outcomes were selected based on the neurodevelopmental domains PAE can affect (Cook et al. 2016) and the measures available within the dataset. The outcomes were measured when the child was aged between 9 months and 8 years and assessed aspects of communication, academic achievement, executive function, and adaptive behaviour. The following measures were included in our analysis of secondary outcomes (Appendix, Table S1):

- SDQ (Goodman 1997)
- Child Behaviour Questionnaire (Very Short Form; CBQ VSF) (Putnam and Rothbart 2006)
- MacArthur–Bates Communication Development Inventory (MacArthur CDI II) (Fenson et al. 2000)
- Adapted Peabody Picture Vocabulary Test (PPVT) (Dunn et al. 1997)
- Stack and topple (Ross 1982)
- Hand Clap task (Neumann et al. 2019)
- Dynamic Indicators Basic Early Literacy Skills (DIBELS) (Good et al. 2002)
- Name and Number Task (Rothman 2005)
- Parent Rating of Oral Language and Literacy (PROLL) (Dickinson et al. 2003).
- B4 School Check (Ministry of Health 2022)
- Social Information Processing (SIP) (Dirks et al. 2014).

### Potential confounders

Based on findings from previous research (Chu et al. 2022), we considered the following maternal baseline characteristics (DCW0) as potential confounding variables in the analysis: age, ethnicity, neighbourhood deprivation, maternal health measures (including smoking status, illicit drug use, and psychological wellbeing), paternal health measures, and home environment (see Appendix, Tables S2–S3).

### Statistical analysis

Statistical analysis was performed using R (the R Project for Statistical Computing, https://www.r-project.org/) via remote access to the secure e-platform hosted by the Growing Up NZ data team. We summarized maternal and paternal characteristics overall and by exposure category, excluding participants where no alcohol exposure data were available. Continuous variables were summarized as the numbers observed and missing, mean, standard deviation (SD), median (Q2), and interquartile range (Q1 and Q3). Categorical variables were summarized as frequencies (*n*) and percentages (%). Missing data were noted but excluded from the analysis.

Between-group differences were tested using the analysis of variance on continuous variables and the chi-square test on categorical variables. We summarized outcomes overall and by maternal alcohol exposure levels using descriptive statistics, and did regression analysis on primary and secondary outcomes from 9 months to 8 years, using linear regression on continuous outcomes and logistic regression on binary outcomes. For each variable, three regression models were considered: an unadjusted model, fully adjusted model including all pre-specified maternal and paternal confounding variables and child sex at birth (see Appendix Table S3 for variables considered in full model), and final adjusted model with stepwise selection including only significant independent covariates in the final model to avoid multicollinearity in selected confounders and test the robustness of the full model. For continuous outcomes, unadjusted and adjusted mean differences between alcohol exposure levels are reported with 95% confidence intervals and *P* values. For binary outcomes, unadjusted and adjusted odds ratios (ORs) are reported with 95% confidence intervals and *P* values. Statistical tests were two sided at the 5% significance level. Widths of confidence intervals have not been adjusted for multiplicity and should not be used in place of hypothesis testing.

### Timing and level of exposure

We did additional analyses to investigate if there was an association between the timing and level of maternal alcohol exposure and the SDQ Total Difficulties Score. Timing of exposure was classified as Early if alcohol was consumed during the first trimester only (T2), as Late if alcohol was consumed after the first trimester only (T3), or as Both if alcohol was consumed throughout the pregnancy (T2 and T3). Level of alcohol exposure was classified as Very low (<1 drink per week), Low (1–3 drinks per week), High (4–19 drinks per week), and Very high (≥20 drinks per week).

### Subgroup analysis

We conducted separate analyses based on self-reported maternal ethnicity for Māori and Pacific mothers and their children, as described for the main analysis.

## Results

### Baseline maternal characteristics

Of the 6822 women enrolled in the study, information about prenatal alcohol use was unavailable for 25 women. Once those with no child and multiple births data were excluded, the baseline sample was 6732 mothers.

The characteristics of the sample by exposure group are described in Tables 1 and 2. Twenty-eight percent of women reported consuming alcohol during their pregnancy. There was a significant difference in the baseline maternal characteristics of the exposure groups for demographic, socioeconomic, and health status variables: The Non-drinker group had a higher proportion of Asian and Pacific women and a higher proportion of women from the most deprived neighbourhoods compared to the Abstainer and Alcohol Exposed groups. A smaller proportion of women in the Abstainer group reported health problems such as asthma, anaemia, and anxiety and depression; however, a greater proportion had diabetes in pregnancy. Compared to the Non-drinker group, a greater proportion of the Alcohol Exposed group were <25 years, self-identified as Māori, had no formal qualifications, and were from the most deprived neighbourhoods. Current smoking status varied between exposure groups; 5.3% of the Non-drinker group reported currently smoking, 9.2% of the Abstainer group, and 17.8% of the Alcohol Exposed group.

**Table 1:** Maternal baseline characteristics by alcohol exposure.

	**Overall**		**Alcohol Exposed**		**Abstainer**		**Non-drinker**		***P* value**
**Variable**	***N***	**%**	***N***	**%**	***N***	**%**	***N***	**%**	
**Total cohort**	6732		1905	(28.3)	2900	(43.1)	1927	(28.6)	
**Maternal age in years, *M* (SD)**	30.05	(5.9)	29.96	(6.3)	30.27	(5.7)	29.82	(5.6)	.024
**Maternal ethnicity**									<.001
NZE	3814	(56.8)	1248	(65.7)	1936	(66.9)	630	(32.7)	
Māori	931	(13.9)	387	(20.4)	351	(12.1)	193	(10.0)	
Pacific	983	(14.6)	191	(10.1)	325	(11.2)	467	(24.3)	
Asian	991	(14.8)	73	(3.8)	282	(9.7)	636	(33.0)	
**Education**									<.001
None	475	(7.1)	195	(10.3)	155	(5.4)	125	(6.5)	
Secondary school	1604	(23.9)	424	(22.3)	623	(21.5)	557	(29.0)	
Diploma	2059	(30.6)	565	(29.8)	899	(31.0)	595	(30.9)	
Bachelor’s degree	1525	(22.7)	411	(21.6)	703	(24.3)	411	(21.4)	
Higher degree	1056	(15.7)	304	(16.0)	516	(17.8)	236	(12.3)	
**Labour status**									<.001
Employed	3630	(56.5)	1072	(58.2)	1726	(62.2)	832	(45.9)	
Unemployed	535	(8.3)	160	(8.7)	168	(6.1)	207	(11.4)	
Student	460	(7.2)	139	(7.6)	201	(7.3)	120	(6.6)	
Not in workforce	1800	(28.0)	470	(25.5)	678	(24.5)	652	(36.0)	
**Current smoker**									<.001
Yes	644	(10.6)	311	(17.8)	243	(9.2)	90	(5.3)	
No	5455	(89.4)	1441	(82.8)	2400	(90.8)	1614	(94.7)	
**Household structure**									<.001
Parent alone	229	(3.4)	89	(4.7)	80	(2.8)	60	(3.1)	
Two parents	4423	(65.7)	1192	(62.6)	2065	(71.2)	1166	(60.5)	
Parent(s) with extended or non-kin	2080	(30.9)	624	(32.8)	755	(26.0)	701	(36.4)	
**Household income**									<0.001
≤$30 K	506	(9.8)	136	(9.3)	157	(6.8)	213	(15.4)	
$30–50 K	724	(14.0)	177	(12.1)	247	(10.7)	300	(21.7)	
$50–70 K	849	(16.5)	185	(12.6)	355	(15.4)	309	(22.3)	
$70–100 K	1190	(23.1)	319	(21.8)	569	(24.7)	302	(21.8)	
>$100–150 K	1886	(36.6)	648	(44.2)	978	(42.4)	260	(18.8)	
**NZ Deprivation Index 2006**									<.001
1–2 (Least deprived)	1091	(16.2)	324	(17.0)	555	(19.1)	212	(11.0)	
3–4	1225	(18.2)	359	(18.9)	597	(20.6)	269	(14.0)	
5–6	1159	(17.2)	328	(17.2)	538	(18.6)	293	(15.2)	
7–8	1410	(21.0)	377	(19.8)	570	(19.7)	463	(24.0)	
9–10 (Most deprived)	1845	(27.4)	515	(27.1)	640	(22.1)	690	(35.8)	

*Notes.* All values are *N* and % unless otherwise stated. Mean (*M*) and standard deviation (SD) reported for age. NZE indicates New Zealand European.

**Table 2:** Maternal baseline health characteristics by alcohol exposure.

	**Overall**		**Alcohol Exposed**		**Abstainer**		**Non-drinker**		***P* value**
**Variable**	***N***	**%**	***N***	**%**	***N***	**%**	***N***	**%**	
**Total cohort**	6732		1905	(28.3)	2900	(43.1)	1927	(28.6)	
**Mother’s health pre-pregnancy: general**									<.001
Poor/fair	684	(10.2)	216	(11.4)	273	(9.42)	195	(10.1)	
Good/very good	4671	(69.4)	1267	(66.6)	2011	(69.4)	1393	(72.4)	
Excellent	1372	(20.4)	420	(22.1)	615	(21.2)	337	(17.5)	
**Health status: depression**									<.001
Never	5576	(82.9)	1493	(78.5)	2392	(82.6)	1691	(87.8)	
Before not during	820	(12.2)	288	(15.1)	378	(13.1)	154	(8.0)	
During	329	(4.9)	122	(6.41)	127	(4.38)	80	(4.2)	
**Health status: anxiety**									<.001
Never	6042	(89.8)	1660	(87.2)	2606	(90.0)	1776	(92.2)	
Before not during	468	(7.0)	175	(9.2)	203	(7.0)	90	(4.7)	
During	216	(3.2)	69	(3.6)	87	(3.0)	60	(3.1)	
**Perceived stress**									.005
Low (≤13)	3283	(53.8)	919	(52.4)	1486	(56.2)	878	(51.5)	
Moderate (14–26)	2676	(43.9)	783	(44.7)	1109	(41.9)	784	(46.0)	
High (≥27)	144	(2.4)	51	(2.9)	49	(1.9)	44	(2.6)	

Overall, 4827 (71.7%) women did not consume any alcohol during pregnancy. Of those women who reported consuming any alcohol during pregnancy (*n* = 1905; 28.3%), most were classified as having had Very Low (46.2%) or Low (30.0%) levels of alcohol exposure (i.e. <1 drink per week or 1–3 drinks per week). Alcohol exposure was classified as Very High (≥20 drinks per week) for only 2.8% and High (4–19 drinks per week) for 21.0% of participants consuming alcohol during pregnancy. Most responses were classified as Early exposure (52.7%), with 20.1% classified as Late exposure and 27.2% as Both.

### Primary outcome: behavioural and emotional development at 8 years

SDQ scores at 8 years were available for 4550 children. The mean Total Difficulties score was 7.55 (SD 4.55); 93.0% of children had Total Difficulties scores that fell in the age-appropriate range, and this was similar across all three exposure groups. There was no significant association between alcohol use in pregnancy and Total Difficulties scores. The adjusted mean difference in Total Difficulties score between Drinkers and Abstainers was −0.034 (95% CI −0.44 to 0.371, *P* = .87).

Most children scored in the age-appropriate range for each of the subscales, with the proportion ranging from 87.6% for Peer Problems to 96.2% for the Prosocial scale, with similar scores across exposure groups. There was no significant association between alcohol use in pregnancy and any of the SDQ subscales. There was no significant association between alcohol use in pregnancy and risk of clinically elevated scores for Total Difficulties score or any of the subscales (Table 3).

**Table 3:** Adjusted model for risk of borderline/abnormal SDQ scores at 8 years.

	**Alcohol Exposed v Abstainer**		**Alcohol Exposed v Non-drinker**		**Abstainer v Non-drinker**	
	**OR**	**95% CI**	**OR**	**95% CI**	**OR**	**95% CI**
**Total difficulties**	0.78	0.52–1.17	0.92	0.58–1.46	1.18	0.80–1.75
**Subscales**						
Emotional problems	1.06	0.74–1.50	1.32	0.88–1.98	1.25	0.87–1.81
Conduct problems	0.81	0.56–1.15	1.00	0.66–1.49	1.23	0.87–1.76
Hyperactivity	0.84	0.61–1.16	1.05	0.73–1.52	1.25	0.91–1.73
Peer problems	0.92	0.68–1.24	1.09	0.78–1.53	1.19	0.89–1.61
Prosocial	0.80	0.49–1.34	1.20	0.65–2.24	1.50	0.89–2.60

### Level and timing of alcohol exposure

There were no significant differences in the Total Difficulties Score or risk of clinically elevated scores by level of alcohol exposure. There were no significant differences in Total Difficulties Score or risk of clinically elevated scores by timing of alcohol exposure.

### Secondary outcomes

We found no significant adverse effects of PAE for any of the secondary outcomes (Table 3 and Appendix Table S4), except with oral language scores at 8 years. Compared to the offspring of mothers who abstained during pregnancy, PROLL scores were higher for offspring of mothers who consumed alcohol in pregnancy (adjusted mean difference (aMD) = 0.67, 95% CI 0.016–0.11, *P* = .0106) indicating better oral language (Table 4). However, there was no significant change in risk of PROLL scores classified as severe (i.e. 2 SD below mean).

**Table 4:** Adjusted model of secondary outcomes.

	**Alcohol Exposed v Abstainer**		**Alcohol Exposed v Non-drinkers**		**Abstainer V Non-drinker**	
	**MD**	**95% CI**	**MD**	**95% CI**	**MD**	**95% CI**
**Behavioural and emotional development**						
SDQ (DCW2)	−0.14	−0.52 to 0.24	−0.32	−0.74 to 0.09	0.85	−0.67 to 1.09
CBQ VSF (DCW5) Surgency	0.02	0.04–0.08	−0.03	−0.10 to 0.03	0.06	−0.12 to 0.00
CBQ VSF (DCW5) Effortful Control	0.03	−0.02 to 0.08	0.02	−0.03 to 0.08	−0.00	−0.05 to 0.04
CBQ VSF (DCW5) Negative Affect	0.01	−0.05 to 0.07	0.02	−0.05 to 0.08	0.01	0.06–0.07
**Communication and adaptive behaviour**						
CDI II (DCW1)	−0.07	−0.33 to 0.18	−0.10	−0.38 to 0.18	−0.03	−0.29 to 0.23
PPVT (DCW5)	0.01	−0.05 to 0.08	−0.04	−0.11 to 0.04	−0.05	−0.11 to 0.02
SIP (DCW8) Aggression-avoidance	−0.05	−0.52 to 0.43	0.20	−0.33 to 0.72	0.24	−0.23 to 0.72
SIP (DCW8) Assertive	−0.23	−0.80 to 0.34	−0.24	−0.86 to 0.39	−0.01	−0.57 to 0.56
**Academic**						
PROLL (DCW5)	0.07*	0.02–0.12	0.06	0.00–0.11	−0.01	−0.06 to 0.04
DIBELS (DCW5)	0.29	−0.55 to 1.13	−0.23	−1.15 to 0.69	−0.52	−1.36 to 0.32
Counting (DCW5)	−0.19	−0.64 to 0.26	−0.02	−0.51 to 0.48	0.17	−0.28 to 0.62
**Executive function**						
Hand Clap (DCW5)	−0.13	−0.52 to 0.27	−0.20	−0.63 to 0.23	−0.08	−0.47 to 0.32

MD: mean difference estimated from the final adjusted regression model with stepwise selection.

### Māori and Pacific subgroup analysis

There were 922 singleton births where women identified as Māori (Appendix Table S5) and 972 singleton births where women identified as Pacific (Appendix Table S6). At baseline, 41.6% Māori mothers and 19.4% Pacific mothers reported consuming alcohol during pregnancy. Of those women who reported consuming alcohol in pregnancy, 63.8% Māori and 78.5% Pacific reported exposure in early pregnancy only, 26.6% Māori and 18.3% Pacific reported exposure in both early and late pregnancy, and 41.1% of Māori and 39.8% of Pacific reported consuming ≥4 drinks per week on average.

Māori did not differ significantly across exposure groups for most baseline characteristics, except the age of participants, smoking status, household structure, and perceived stress. Compared to the other groups, a larger proportion of women in the Alcohol Exposed group were <25 years of age, current smokers, and parenting alone, and a smaller proportion reported low levels of perceived stress.

For the children of Māori women with SDQ scores available (*n* = 524), we found no significant difference in the Total Difficulties score at 8 years by alcohol exposure (Alcohol Exposed v Abstainer aMD = −0.099, 95% CI −1.364 to 1.165, *P* = .877; Alcohol Exposed v Non-drinker aMD = 0.325, 95% CI −1.052 to 1.702, *P* = .6426). Children in the Māori subgroup who were prenatally exposed to alcohol were at increased risk of clinically elevated scores on the Emotional Problems scale (OR = 4.336, 95% CI 1.118–21.995, *P* = .468) and Peer Problems (OR = 2.319, 95% CI 1.061–5.293, *P* = .039) relative to non-drinkers. There were no significant differences between Alcohol Exposed and Abstainer categories. Children in the Māori whose mothers abstained in pregnancy were at increased risk of Peer Problems compared to non-drinkers (OR = 2.595, 95% CI 1.277–5.601, *P* = .011; see Appendix Table S7).

There were significant differences across exposure groups for Pacific mothers for the following baseline characteristics: maternal age, education status, smoking status, household structure, and household income. Compared to Non-drinker and Abstainer groups, a larger proportion of the Alcohol Exposed group were <25, reported no formal qualification, and parented alone; and compared to Abstainer, a greater proportion of Alcohol Exposed and Non-drinker had a household income of ≤$30 000.

For the children of Pacific women with SDQ scores available (*n* = 390), there was no significant difference in the Total Difficulties score at 8 years by alcohol exposure category for Pacific women (Alcohol Exposed v Abstainer aMD = −0.417, 95% CI −2.018 to 1.184, *P* = .442). For Pacific women, there was no significant increased risk in clinically elevated scores for Total Difficulties score (OR = 0.715, 95% CI 0.205–2.198, *P* = .13) or for any of the SDQ subscales.

## Discussion

### Main findings

We found no associations between PAE and neurodevelopmental outcomes in this representative cohort of NZ children at 8 years of age. For children of Māori mothers, PAE was associated with an increased risk of emotional and peer problems. PAE was associated with higher scores on the parent-related oral language, indicating improved oral language.

### Interpretation

The lack of relationship between PAE and behavioural and emotional development is consistent with some studies (D'souza et al. 2019), but is contrary to research that suggests that PAE had detrimental effects on behavioural and emotional development (Skogerbo et al. 2013; Schoeps et al. 2018; Chu et al. 2022). PAE can result in FASD, where people have significant impairments in three or more neurocognitive domains (Cook et al. 2016). Some individuals with FASD experience difficulties with behavioural and emotional development. If individuals with FASD are left unsupported, they can face challenges throughout life (Streissguth et al. 2004). Therefore, it is vital to understand the consequences of PAE and provide clear messaging on PAE to the general public, particularly mothers.

The lack of relationship could be due to several methodological factors, including the measures available in the dataset, confounding variables, and measure of PAE. It is likely that there are other pre- and postnatal variables besides PAE influencing the relationship between PAE and neurodevelopmental outcomes. We controlled for those where data were available, such as tobacco use and neighbourhood deprivation, but no relationship was found. Therefore, other confounding variables, particularly those in the postnatal environment that are related to children’s development, such as traumatic childhood events (Price et al. 2017) and quality of parent–child relationship (Pinquart 2017), which can also effect children’s neurodevelopment domains, were not controlled for.

Our measures of PAE may have been insufficiently accurate. PAE and FASD are highly stigmatized (Davis et al. 2010) potentially resulting in mothers underreporting their PAE. Therefore, children in the Non-drinker or Abstainer group may have been exposed to alcohol and those who were exposed to alcohol may have been exposed to larger amounts, masking the effect of PAE on neurodevelopmental outcomes. Surprisingly, we found no association between high levels of PAE and behavioural and emotional development, despite strong evidence to suggest detrimental effects of large or binge PAE (Skogerbo et al. 2013, Schoeps et al. 2018, Chu et al. 2022). Perhaps this could be due to less than 6% of mothers reporting high or very high alcohol consumption in our study sample. In addition, the amount of PAE was measured on one item, so it could be that mothers took an average amount across the pregnancy rather than reporting binge episodes. Therefore, future research needs to control for postnatal confounding variables and consider how PAE is measured.

We are unsure why Māori children were more likely to have emotional and peer problems, but no effect was found for the overall sample. In this sample, Māori mothers reported higher rates of alcohol-exposed pregnancies and consuming larger amounts of alcohol than the total sample, which could reflect the greater burden of PAE for Māori women (Walker 2019). This reporting pattern could have made it easier to detect the effect of PAE on outcomes. Alternately, other postnatal confounding factors that were more common in Māori women, such as parental smoking and parenting alone, contribute to the increased emotional and peer problems for children. The complexity of this relationship is compounded by the lack of standardized SDQ data in NZ. Māori children in general might have higher scores on emotional and peer problems, due to the SDQ scale, which may have low cross-cultural utility (Kersten et al. 2016). Therefore, it is unclear if the relationship between PAE and behavioural and emotional development for Māori children is due to PAE, other confounding variables, or inappropriate measurement. Regardless, these findings highlight the importance of investigating the effect of PAE in NZ, and the need for non-stigmatizing interventions, particularly for Māori women and children.

### Strengths and limitations

The GUiNZ study provides access to a diverse and representative cohort of NZ children and their families. The prospective nature of the GUiNZ study allows several variables to be controlled for in analysis. However, PAE was measured on an average estimate of consumption across pregnancy, and was effected by stigma (Davis et al. 2010) or recall bias. Therefore, an accurate understanding of PAE may not have been captured particularly for those with episodes of binge drinking. Likewise, the developmental outcomes assessed, and how they were measured, are limited to those that are routinely measured within the GUiNZ study. The SDQ, despite being widely used in PAE studies, is a self-report questionnaire completed by parents or often teachers. There has been some evidence that there can be poor agreement when multiple informants complete the SDQ for the same child (Vaz et al. 2016), indicating that the SDQ is subject to context and observer biases. Therefore, other psychometric tests of behavioural and emotional development may be useful.

In addition, the SDQ may not be a good fit for all cultural contexts, including Māori. Due to the lack of NZ norms, the current study used Australian norms to classify SDQ scores. In particular, the SDQ subscales have shown poor consistency across cultures, while the Total Difficulties Score show relatively good validity (Morton et al. 2012). Likewise, other measures of the secondary outcomes may need to be explored to understand the effect of PAE.

Within the available dataset, information was not available if children had been diagnosed with FASD, a developmental disability, or another alcohol-related development disorder. Individuals with FASD have significant impairment on at least 3 of the10 neurodevelopmental domains that can show impairment for children with FASD (Cook et al. 2016). Due to the wide variability in developmental outcomes and impairments with PAE, not every child with FASD will show difficulties with behavioural and emotional development. If FASD diagnostic information is available within a dataset, then the neurodevelopmental outcomes between those with FASD and those without can be compared However, given the difficulties in accessing a diagnosis in NZ, until people can easily access a diagnosis, this may not be possible.

To better understand the effects of PAE in NZ, data must come from a study designed for that purpose. Although the GUiNZ study provides invaluable data in exploring outcomes, it was not designed with the intention of solely studying PAE or FASD, so diverse and important measures were not included. As research has developed and we have been able to better understand confounding variables that can moderate the effect of PAE, it is important to have a study design that can control and collect these measures as they occur. Therefore, our study highlights the importance of considering the dataset that information is drawn from and the variables that are studied.

## Conclusion

This study did not find an association between PAE and behavioural and emotional development of children aged 8 years on a range of screening instruments. However, this is more likely to reflect underreporting of PAE, limitations in the dataset available, or confounding variables, rather than an absence of PAE impact on developmental outcomes. Given the weight of the evidence of the detrimental effects of PAE on a range of neurodevelopmental domains, including behavioural and emotional development, it is likely that PAE has negative effects on development for NZ children. Concerningly, 28% of pregnancies in this study were exposed to some amount of alcohol, highlighting the need for clear consistent messages around alcohol consumption to the public in NZ. If recognition of PAE and FASD is not accepted at the government and healthcare level, mothers will continue to be given conflicting messages or be confused about safe levels of PAE.

## Supplementary Material

Supplementary_Table_S1_v3_agae029

Supplementary_Table_S2_agae029

Supplementary_Table_S3_v2_agae029

Supplementary_Table_S4_v2_agae029

Supplementary_Table_S5_agae029

Supplementary_Table_S6_agae029

Supplementary_Table_S7_agae029

## Data Availability

Data access is limited due to the Data Assess Agreement for accessing the GUiNZ cohort data.
