# Supplementary material for: Investigating the relationship between prenatal alcohol exposure and children’s behavioural and emotional development: analysis of the Growing Up in New Zealand study
Source: Alcohol Alcohol. 2024 Apr 27;59(3):agae029. doi: 10.1093/alcalc/agae029 (PMC11055961; doi:10.1093/alcalc/agae029)
Supplement: Supplementary_Table_S1_v3_agae029 [file supplementary_table_s1_v3_agae029.docx]

#### Supplementary Table S1: Measures of primary and secondary outcomes

| **Variable** | | **Description** |  |
| --- | --- | --- | --- |
| Primary Outcome | | | |
| Behavioural and emotional development | | SDQ Total Difficulties score, generated by summing all 25 items (excluding the prosocial items) and can range from 0 to 40, with more extreme scores indicating more difficulties (DCW 8) | |
| SDQ as a dichotomised variable | | Scores categorised as either normal (0-13) or borderline/abnormal (14-40) based on cut-offs taken from Australian norms.^[[1]](#footnote-1)^ | |
| Subscales | Emotional problems | Worried, unhappy, or easily scared | |
|  | Conduct problems | Loses temper, argumentative, fights with other children | |
|  | Hyperactivity | Restless, easily distracted | |
|  | Peer problems | Solitary, not liked by other children | |
|  | Prosocial | Considerate, shares with others | |
| Secondary Outcomes | | | |
| Behavioural and emotional development | | SDQ (DCW2). | |
|  |  | Child Behaviour Questionnaire (Very Short Form; CBQ VSF), a measure of temperament administered to parents (DCW5). | |
| Language | | MacArthur-Bates Communication Development Inventory (MacArthur CDI II) – First communication Gestures Scale (12-items), a measure of non-verbal communication administered to parents (DCW1). | |
|  |  | Adapted Peabody Picture Vocabulary Test (PPVT), a test of receptive vocabulary administered by child observation (DCW5). A derived variable – latent receptive language – was generated from the core 20 items. | |
| Executive function | | Stack and topple, an observation of different types of attention administered in the child observation (DCW2). | |
|  |  | Hand Clap task, a test of response inhibition consisting of 16 trials administered in the child observation (DCW5). | |
| Academic achievement | | Dynamic Indicators Basic Early Literacy Skills (DIBELS), a measure of reading an early literacy skill administered to children (DCW5). | |
|  |  | Name and Number Task, a measure of academic skill taken form the Who Am I? Developmental Assessment (LSAC) administered to children (DCW5). | |
|  |  | Parent Rating of Oral Language and Literacy (PROLL), a measure of oral language administered to parents (DCW5). | |
|  |  | B4 school check, self-reported areas of concern from parents (DCW6). | |
| Adaptive behaviour, social skills, and communication | | Social Information Processing, a measure adapted from the Peer Provocation Inventory (DWC8) | |

No New Zealand norms of the SDQ have been published although the Ministry of Health guidance for the B4 School check includes thresholds for secondary assessment and referral.

1. [↑](#footnote-ref-1)
