# Supplementary material for: Investigating the relationship between prenatal alcohol exposure and children’s behavioural and emotional development: analysis of the Growing Up in New Zealand study
Source: Alcohol Alcohol. 2024 Apr 27;59(3):agae029. doi: 10.1093/alcalc/agae029 (PMC11055961; doi:10.1093/alcalc/agae029)
Supplement: Supplementary_Table_S2_agae029 [file supplementary_table_s2_agae029.docx]

#### Supplementary Table S2: Potential confounders

| Domain | Measure |
| --- | --- |
| Maternal baseline characteristics (DCW0) | Age (years) at antenatal interview |
|  | Self-prioritised maternal ethnicity (Statistics New Zealand Level 1 classification) |
|  | Pre-pregnancy weight (kg), height (m) and BMI (kg/m2) |
|  | Highest completed qualification |
|  | Labour force status (i.e., employed, unemployed, student, not in workforce) |
|  | Current smoking status (i.e., current smoker or non-smoker) |
|  | Medication uses during pregnancy |
|  | General health status, disability, and clinical diagnoses |
|  | Edinburgh postnatal depression scale (Normal [0-11], Abnormal [12 or above]) (38) |
|  | Perceived stress scale (0-40) |
|  | Interparental relationship |
|  | Household structure (i.e., parent alone, two parents, parent(s) with extended family, parent(s) living with non-kin |
|  | Total annual household income (≤$20K - >$150K) |
|  | NZ Deprivation Index 2006 |
|  | Paternal alcohol and tobacco use |
| Outcomes collected in contemporaneous data waves | Current NZ Deprivation Index 2006 or 2013 |
|  | Maternal drug use at 9 months |
|  | Maternal mental Health (PHQ) (39) |
|  | Chaos, Hubbub and Order Scale (CHAOS) (40) |

# 
