# Supplementary material for: Investigating the relationship between prenatal alcohol exposure and children’s behavioural and emotional development: analysis of the Growing Up in New Zealand study
Source: Alcohol Alcohol. 2024 Apr 27;59(3):agae029. doi: 10.1093/alcalc/agae029 (PMC11055961; doi:10.1093/alcalc/agae029)
Supplement: Supplementary_Table_S3_v2_agae029 [file supplementary_table_s3_v2_agae029.docx]

#### Supplementary Table S3: Confounding variables include in stepwise model at each data collection wave (DCW)

| **Data collection wave** | **Outcomes** | **Confounding Variables** |
| --- | --- | --- |
| DCW8 | SDQ: Total Difficulties, Emotional Problems, Conduct Problems, Hyperactivity, Peer Problems, Prosocial; Social Information Processing: Aggression-Avoidance, Assertive | Sex; Ethnicity (child); Weight at birth; Maternal prenatal smoking status; Maternal depression at 8 years; Household environment at 8 years; Neighbourhood deprivation at 8 years |
| DCW1 | Weight: Birth, 6 weeks, 9 months; Preterm; MacArthur-Bates CDI II | Maternal age; maternal ethnicity; maternal education; maternal labour status; maternal prenatal smoking status; household structure; household income; neighbourhood deprivation; general health during pregnancy; anxiety during pregnancy; depression during pregnancy; postnatal depression risk; interparental relationship; maternal drug use at 9 months; paternal smoking status; sex; Term; Weight at birth |
| DCW2 | Stack and Topple task; SDQ: Total Difficulties | Sex; Weight at birth; Ethnicity; maternal prenatal smoking status; neighbourhood deprivation at 2 years |
| DCW5 | PPVT (derived); Luria test; DIBELS; PROLL; Child Behaviour Questionnaire: Surgency, Negative Affect, Effortful Control | Sex; Weight at birth; Ethnicity; maternal prenatal smoking status; maternal depression at 5 years; neighbourhood deprivation at 5 years |
| DCW6 | B4School Check: Learning difficulties, behaviour, mobility, speech, no concerns | Sex; Weight at birth; Ethnicity; maternal prenatal smoking status; maternal depression at 5 years; neighbourhood deprivation at 6 years |
| DCW8 | SDQ; SIP | Offspring sex; self-identified ethnicity; weight at birth; maternal smoking status during pregnancy; maternal mental health at 8 years; neighbourhood deprivation at 8 years; household chaos at 8 years |
