# Supplementary material for: Investigating the relationship between prenatal alcohol exposure and children’s behavioural and emotional development: analysis of the Growing Up in New Zealand study
Source: Alcohol Alcohol. 2024 Apr 27;59(3):agae029. doi: 10.1093/alcalc/agae029 (PMC11055961; doi:10.1093/alcalc/agae029)
Supplement: Supplementary_Table_S4_v2_agae029 [file supplementary_table_s4_v2_agae029.docx]

#### Supplementary Table S4: Logistic regression stepwise adjusted model of secondary outcomes

|  | **Alcohol Exposed v Abstainer** | | **Alcohol Exposed v Non-Drinkers** | | **Abstainer V Non-Drinker** | |
| --- | --- | --- | --- | --- | --- | --- |
|  | **OR** | **95% CI** | **OR** | **95% CI** | **OR** | **95% CI** |
| **Behavioural and emotional development** |  |  |  |  |  |  |
| SDQ (DCW2) | 0.85 | 0.67-1.09 | 1.01 | 0.78-1.29 | 0.86 | 0.66- 1.12 |
| **Academic** |  |  |  |  | 1.06 | 0.77- 1.46 |
| PROLL (DCW5) Severe | 0.91 | 0.65-1.26 | 1.00 | 0.67-1.38 | 1.09 | 0.63- 1.95 |
| B4 School (DCW6) Learning Difficulties | 0.87 | 0.48-1.53 | 0.95 | 0.50-1.80 | 1.04 | 0.64- 1.71 |
| B4 School (DCW6) Behaviour | 1.00 | 0.61-1.61 | 1.03 | 0.60-1.78 | 1.24 | 0.47- 3.62 |
| B4 School (DCW6) Mobility | 0.80 | 0.27-2.11 | 0.99 | 0.31-3.19 | 1.22 | 0.83- 1.83 |
| B4 School (DCW6) Speech | 0.93 | 0.63-1.36 | 1.14 | 0.73-1.77 | 0.83 | 0.57- 1.21 |
| **Executive Function** |  |  |  |  | 0.94 | 0.78- 1.12 |
| Stack and Topple (DCW2) Joint Attention | 0.93 | 0.65-1.32 | 0.77 | 0.51-1.32 | 1.01 | 0.81- 1.25 |
| Stack and Topple (DCW2) Inhibitory Control | 1.16 | 0.96-1.39 | 1.09 | 0.89-1.33 | 0.86 | 0.66- 1.12 |
| Stack and Topple (DCW2) Sustained Attention | 1.17 | 0.93-1.46 | 1.18 | 0.92-1.51 | 1.06 | 0.77- 1.46 |
