# Supplementary material for: Investigating the relationship between prenatal alcohol exposure and children’s behavioural and emotional development: analysis of the Growing Up in New Zealand study
Source: Alcohol Alcohol. 2024 Apr 27;59(3):agae029. doi: 10.1093/alcalc/agae029 (PMC11055961; doi:10.1093/alcalc/agae029)
Supplement: Supplementary_Table_S5_agae029 [file supplementary_table_s5_agae029.docx]

#### Supplementary Table S5: Baseline characteristics of Māori mothers by exposure group

|  | **Alcohol Exposed**  **(N=387)** | | **Abstainer**  **(N=351)** | | **Non-Drinker**  **(N=193)** | | **p-value** |
| --- | --- | --- | --- | --- | --- | --- | --- |
| **Variable** | **N** | **%** | **N** | **%** | **N** | **%** |  |
| **Age Mean (SD)** | 26.46 | (6.5) | 27.23 | (6.0) | 28.77 | (6.5) | <0.001 |
| **Education** |  | |  | |  | | 0.54 |
| None | 92 | (23.8) | 60 | (17.1) | 35 | (18.1) |  |
| Secondary School | 105 | (27.2) | 106 | (30.3) | 50 | (25.9) |  |
| Diploma | 137 | (35.5) | 126 | (36.0) | 70 | (36.3) |  |
| Bachelor's Degree | 36 | (9.3) | 46 | (13.1) | 26 | (13.5) |  |
| Higher Degree | 16 | (4.2) | 12 | (3.4) | 12 | (6.2) |  |
| **Labour Status** |  | |  | |  | | 0.23 |
| Employed | 132 | (35.5) | 144 | (43.0) | 64 | (35.0) |  |
| Unemployed | 59 | (15.9) | 42 | (12.5) | 20 | (10.9) |  |
| Student | 39 | (10.5) | 46 | (13.7) | 25 | (13.7) |  |
| Not in Workforce | 142 | (38.2) | 103 | (30.8) | 74 | (40.4) |  |
| **Current Smokers** | 151 | (43.1) | 94 | (29.8) | 35 | (20.6) | <0.001 |
| **Household Income** |  | |  | |  | | 0.56 |
| <=$30K | 56 | (22.7) | 46 | (18.9) | 22 | (15.4) |  |
| $30-50K | 60 | (23.3) | 32 | (13.2) | 30 | (21.0) |  |
| $50-70K | 41 | (15.9) | 45 | (18.5) | 27 | (18.9) |  |
| $70-100K | 57 | (22.1) | 56 | (23.1) | 37 | (25.9) |  |
| >$100-150K | 44 | (15.1) | 64 | (26.3) | 27 | (18.9) |  |
| **Neighbourhood deprivation (NZDEP)** |  | |  | |  | | 0.38 |
| 1-2 (Least deprived) | 18 | (4.7) | 29 | (8.3) | 8 | (4.2) |  |
| 3-4 | 31 | (8.0) | 38 | (10.8) | 20 | (10.4) |  |
| 5-6 | 43 | (11.1) | 46 | (13.1) | 32 | (16.6) |  |
| 7-8 | 95 | (24.6) | 75 | (21.4) | 48 | (24.9) |  |
| 9-10 (Most deprived) | 200 | (51.7) | 163 | (46.4) | 85 | (44.0) |  |
| **Mother's Health Pre-pregnancy: General** |  | |  | |  | | 0.084 |
| Poor | 25 | (6.5) | 21 | (6.0) | 5 | (2.6) |  |
| Fair | 68 | (17.6) | 67 | (19.1) | 24 | (12.5) |  |
| Good | 156 | (40.3) | 118 | (33.6) | 69 | (35.9) |  |
| Very Good | 87 | (22.5) | 106 | (30.2) | 59 | (30.7) |  |
| Excellent | 51 | (13.2) | 39 | (11.1) | 35 | (18.2) |  |
