# Supplementary material for: Investigating the relationship between prenatal alcohol exposure and children’s behavioural and emotional development: analysis of the Growing Up in New Zealand study
Source: Alcohol Alcohol. 2024 Apr 27;59(3):agae029. doi: 10.1093/alcalc/agae029 (PMC11055961; doi:10.1093/alcalc/agae029)
Supplement: Supplementary_Table_S6_agae029 [file supplementary_table_s6_agae029.docx]

#### Supplementary Table S6: Baseline characteristics of Pacific mothers by exposure group

|  | **Alcohol Exposed (N=191)** | | **Abstainer (N=325)** | | **Non-Drinker (N=467)** | | **p-value** |
| --- | --- | --- | --- | --- | --- | --- | --- |
| **Variable** | **N** | **%** | **N** | **%** | **N** | **%** |  |
| **Age Mean (SD)** | 26.39 | (6.4) | 27.14 | (6.2) | 29.36 | (6.2) | <0.001 |
| **Education** |  | |  | |  | | <0.001 |
| None | 42 | (22.2) | 35 | (10.8) | 45 | (9.7) |  |
| Secondary School | 62 | (32.8) | 128 | (39.5) | 233 | (50.0) |  |
| Diploma | 62 | (32.8) | 121 | (37.4) | 159 | (34.1) |  |
| Bachelor's Degree | 14 | (7.4) | 27 | (8.3) | 24 | (5.2) |  |
| Higher Degree | 9 | (4.8) | 13 | (4.0) | 5 | (1.1) |  |
| **Labour Status** |  | |  | |  | | 0.11 |
| Employed | 80 | (44.2) | 138 | (45.4) | 148 | (34.3) |  |
| Unemployed | 35 | (19.3) | 45 | (14.8) | 74 | (17.1) |  |
| Student | 7 | (3.9) | 18 | (5.9) | 25 | (5.8) |  |
| Not in Workforce | 59 | (32.6) | 103 | (33.9) | 185 | (42.8) |  |
| **Current Smokers** | 52 | (29.9) | 60 | (21.1) | 18 | (4.5) | <0.001 |
| **Household Income** |  | |  | |  | | 0.0016 |
| <=$30K | 25 | (21.2) | 35 | (16.3) | 74 | (25.4) |  |
| $30-50K | 29 | (24.6) | 45 | (20.9) | 90 | (30.9) |  |
| $50-70K | 20 | (17.0) | 45 | (20.9) | 61 | (21.0) |  |
| $70-100K | 15 | (12.7) | 44 | (20.5) | 44 | (15.1) |  |
| >$100-150K | 29 | (24.6) | 46 | (21.4) | 22 | (7.5) |  |
| **Neighbourhood deprivation (NZDEP)** |  | |  | |  | | 0.059 |
| 1-2 (Least deprived) | 1 | (0.5) | 10 | (3.1) | 7 | (1.5) |  |
| 3-4 | 16 | (8.4) | 15 | (4.6) | 13 | (2.8) |  |
| 5-6 | 10 | (5.2) | 27 | (8.3) | 22 | (4.7) |  |
| 7-8 | 36 | (18.9) | 68 | (20.9) | 97 | (20.8) |  |
| 9-10 (Most deprived) | 128 | (67.0) | 205 | (63.1) | 328 | (70.2) |  |
| **Mother's Health Pre-pregnancy: General** |  | |  | |  | | 0.29 |
| Poor | 13 | (6.8) | 17 | (5.2) | 16 | (3.4) |  |
| Fair | 32 | (16.8) | 39 | (12.0) | 59 | (12.7) |  |
| Good | 95 | (49.7) | 155 | (47.7) | 209 | (44.9) |  |
| Very Good | 34 | (17.8) | 68 | (20.9) | 102 | (21.9) |  |
| Excellent | 17 | (8.9) | 46 | (14.2) | 80 | (17.2) |  |
