# Supplementary material for: Investigating the relationship between prenatal alcohol exposure and children’s behavioural and emotional development: analysis of the Growing Up in New Zealand study
Source: Alcohol Alcohol. 2024 Apr 27;59(3):agae029. doi: 10.1093/alcalc/agae029 (PMC11055961; doi:10.1093/alcalc/agae029)
Supplement: Supplementary_Table_S7_agae029 [file supplementary_table_s7_agae029.docx]

#### Supplementary Table S7: Logistic regression stepwise adjusted model of SDQ in Māori mother’s subgroup

|  | **Abstainer V Non-Drinker** | | | **Alcohol Exposed v Non-Drinkers** | | | **Alcohol Exposed v Abstainer** | | |
| --- | --- | --- | --- | --- | --- | --- | --- | --- | --- |
|  | **OR** | **95% CI** | **p** | **OR** | **95% CI** | **p** | **OR** | **95% CI** | **p** |
| Total Difficulties | 2.08 | 0.84-5.58 | 0.13 | 2.27 | 0.81-6.73 | 0.12 | 1.09 | 0.46-2.54 | 0.84 |
| **Subscales** |  |  |  |  |  |  |  |  |  |
| Emotional Problems | 3.38 | 0.98-15.95 | 0.08 | 4.34 | 1.12-22.00 | 0.05* | 1.28 | 0.46-3.50 | 0.63 |
| Conduct Problems | 0.95 | 0.45-2.05 | 0.90 | 0.99 | 0.43-2.31 | 1.00 | 1.04 | 0.47-2.28 | 0.92 |
| Hyperactivity | 1.00 | 0.45-2.27 | 0.99 | 0.87 | 0.34-2.19 | 0.77 | 0.87 | 0.36-2.01 | 0.74 |
| Peer Problems | 2.60 | 1.28-5.60 | 0.01* | 2.32 | 1.06-5.29 | 0.04* | 0.89 | 0.47-1.67 | 0.73 |
| Prosocial | 7.90 | 1.37-150.86 | 0.06 | 2.04 | 0.08-55.73 | 0.63 | 0.26 | 0.01-1.60 | 0.22 |
